# Characterization of Wet and Dry Deposition in the Downwind of Industrial Sources in a Dry Tropical Area

**DOI:** 10.1100/tsw.2001.388

**Published:** 2001-12-19

**Authors:** Raj K. Singh, Raj M. Agrawal

**Affiliations:** Department of Botany, Banaras Hindu University, Varanasi, India

**Keywords:** atmospheric dry and wet depositions, trace gases, power plant emission

## Abstract

An atmospheric deposition study was conducted in the downwind of Shaktinagar Thermal Power Plant (STPP), Renusagar Thermal Power Plant (RTPP), and Anpara Thermal Power Plant (ATPP), at Singrauli region, Uttar Pradesh (UP), India to characterize dry and wet deposition in relation to different pollution loading. During the study period, dry and wet depositions and levels of gaseous pollutants (SO2 and NO2) were estimated across the sites. Dry deposition was collected on a monthly basis and wet deposition on an event basis. Depositions were analyzed for pH, nitrate (NO3-), ammonium (NH4+), and sulphate (SO4(2-)) contents. Dry deposition rate both collected as clearfall and throughfall varied between 0.15 to 2.28 and 0.33 to 3.48 g m(-2) day(-1), respectively, at control and maximally polluted sites. The pH of dry deposition varied from 5.81 to 6.89 during winter and 6.09 to 7.02 during summer across the sites. During the rainy season, the mean pH of clear wet deposition varied from 6.56 to 7.04 and throughfall varied from 6.81 to 7.22. The concentrations of NO2 and SO2 pollutants were highest during the winter season. Mean SO2 concentrations varied from 18 to 75 g m(-3) at control and differently polluted sites during the winter season. The variation in NO2 concentrations did not show a pattern similar to that of SO2. The highest NO2 concentration during the winter season was 50 g m(-3), observed near RTPP. NO2 concentration did not show much variation among different sites, suggesting that the sources of NO2 emission are evenly distributed along the sites. The concentrations of NH4+, NO3-, and SO4(2-) ions in dry deposition were found to be higher in summer as compared to the winter season. In dry deposition (clearfall) the concentrations of NH4+, NO3-, and SO4(2-) varied from 0.13 to 1.0, 0.81 to 1.95, and 0.82 to 3.27 mg l(-1), respectively, during winter. In wet deposition (clearfall), the above varied from 0.14 to 0.74, 0.81 to 1.82, and 0.67 to 2.70 mg l(-1), respectively. The study clearly showed that both dry and wet depositions varied between the sites and season, suggesting significant impact of industrial activities in modifying the atmospheric input. The nonacidic deposition suggests that there is no threat of acidification of the receiving ecosystem at present.

## INTRODUCTION

Rapid industrialization and urbanization are causing serious concern about environmental pollution. The problem of air pollution has assumed serious proportions in the metropolitan cities and around industrial areas. Air pollution is steadily increasing because of spiraling fossil fuel consumption by large fossil-fuelled, electricity-generating plants and the transport sector. Fossil fuel consumption in India has increased from 75 Mt $year^{-1}$ in 1964 to 245 Mt $year^{-1}$ in 1990[1]. It is well established that stresses from regionally transported gaseous pollutants and acidic deposition can cause structural and functional changes in plant communities[2], leading to subtle degradation of ecosystems[3,4].

Depositional processes also regulate the chemical composition of the atmosphere. The possible ways by which these depositions may alter the nutrient cycling in ecosystem are either by altering the availability of trace species in the soil or by atmospheric addition or depletion of different nutrients in foliage[5]. Several studies have reported on the deposition of nutrients and heavy metals in different ecosystem components around pollugenic sources in developed countries[6,7,8]. In Europe and North America, emissions of sulphur and nitrogen oxides into the atmosphere have caused widespread environmental effects, including a downward trend in the pH of precipitation; acidification of soils, surface water, and groundwater; injury to vegetation; and corrosion of building materials[9]. Acid deposition has been proposed as the major factor associated with forest decline in Europe and in the northeastern U.S.[10]. High rates of deposition of sulphate ($SO_4^{2-}$), nitrate ($NO_3^-$), and ammonium ($NH_4^+$) may lead to a depletion of base cations in surface soil and phytotoxic concentrations of heavy metals[11].

In Asian countries air pollutant emissions are rapidly increasing due to the high rate of industrialization and energy production[12,13,14,15]. In northeast Asia, air pollution levels are exceeding World Health Organization (WHO) ambient standards[16,17]. India is a quickly developing economy where air pollution levels are continuously rising in metropolitan cities and industrial regions[18,19]. Events of acid precipitation were also reported from some parts of the country[20,21,22]. An abundance of alkaline substances in the atmosphere is, however, found to reduce the acidity of wet depositions[23]. The present study was undertaken to estimate the spatial and temporal variations in gaseous pollutants and $NO_3^-$, $NH_4^+$, and $SO_4^{2-}$ concentrations and pH of dry and wet depositions in industrial region of Sonbhadra districts, Uttar Pradesh (UP), India.

## MATERIALS AND METHODS

### Study Area

The study was conducted between the latitudes of 24°- 24° 12′ 31″N and longitudes of 82° 40′ – 82° 44′ 30″ E in the Sonbhadra district of UP. The general topography of the area is undulating with hills, valleys, and flats interspersed. The elevation ranges from 200 to 450 m above mean sea level. There are 6 thermal power plants and 11 open cast coal mines in the area. The construction and operation of thermal power plants and coal mines have led to serious air, water, and soil pollution and a land degradation and deforestation problem[24,25].

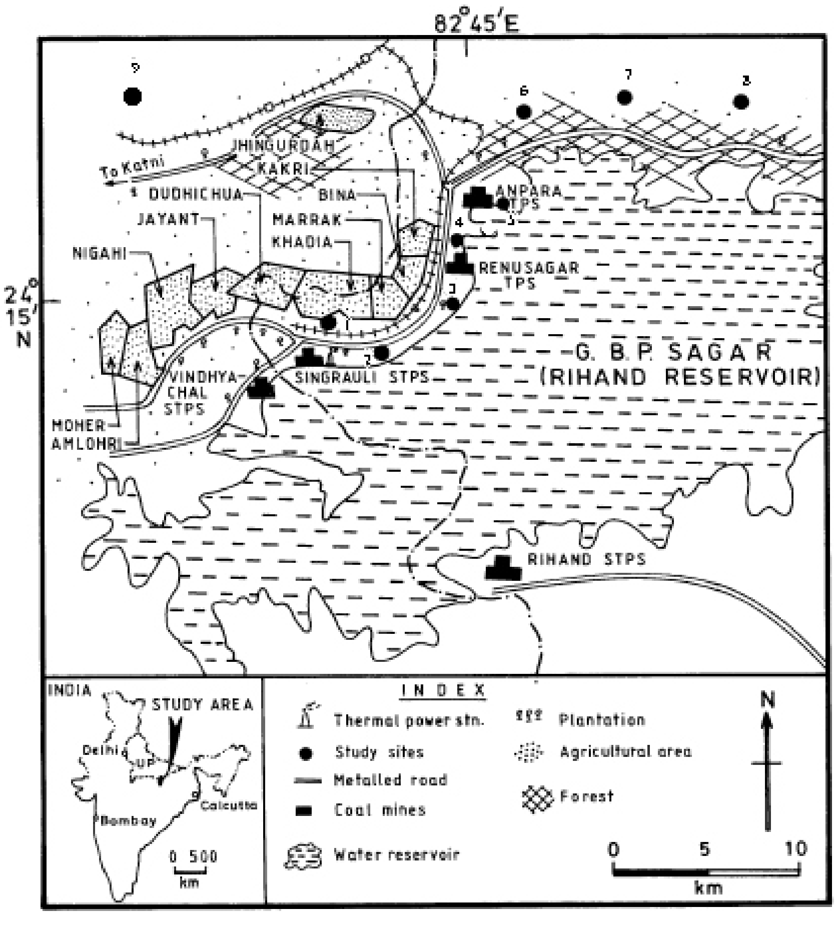

**FIGURE 1.** Map of Research Sites.

The climate of the region is dry tropical; the total rainfall was 1234 mm, out of which 95% occurred from June to September in rainy season. During the study period, the mean monthly maximum temperature varied from 21.2 to 41.3°C and the mean monthly minimum temperature from 13.5 to 34.4°C. The predominant wind direction during the study was N – NE and wind speed varied from 7.5 to 8.9 km $h^{-1}$. The maximum humidity varied from 52 to 90% and the minimum humidity varied from 27 to 84%. The present study was confined in the area affected mainly by Shaktinagar Thermal Power Plant (STPP, 2000 MW), Renusagar Power Plant (RTPP, 285 MW) and Anpara Thermal Power Plant (ATPP, 2000 MW), and Bina and Kakari open cast coal mines emissions.

Nine sites were selected in the leeward side of STPP on a 25-km transect passing through RTPP and ATPP considering the vegetation, physical relief, and undulations. Table 1 describes the characteristics of the sites.

## Sampling and Analysis

### *Air Quality Monitoring*

Ambient air quality monitoring for suspended particulate matter (SPM), $SO_2$, and $NO_2$ were done in 10-day intervals on each study site. Total SPM was collected on glass fiber filter paper by using a high volume air sampler (Model 410 Envirotech, India) operated continuously for 24 h. The $SO_2$ concentration was monitored through an $SO_2$ analyzer (Model 319, Kimoto, Japan). The $SO_2$ concentration was also monitored through a handy air sampler after absorbing a known amount of air in a 0.1 M sodium tetrachloromercurate solution using the method described by West and Gaeke[26]. $NO_2$ concentration was measured by absorbing the gas in 0.01 N NaOH and 1% sodium arsenite, which was later analyzed colorimetrically[27]. The samples were collected for 24 h in 10-day intervals; each time three replicates were taken.

## Atmospheric Depositions

Dry deposition samples were collected on a monthly basis using a plastic funnel fitted on a 2-l polyethylene bottle, previously rinsed with deionised water following the method of Harrison[28]. The funnel was mounted 3 m above the ground level at all the sites. The plastic container was exposed throughout the summer and winter seasons. The measurement of dry deposition of air pollution is always a challenging task. No one method has gained universal acceptance. It is true that surrogate surfaces, as in the present case, are inadequate in simulating natural surfaces[29], but these are the only possible way to provide for routine monitoring and chemical analysis[30]. Gaseous adsorption and aerosol impaction are not measured fully through this method. This method is, however, variously used for dry deposition measurement by Indian scientists[30,31,32]. Plastic bottles were placed in an open area for the collection of clearfall and under the canopy of trees for measurement of throughfall deposition. Dry deposition of throughfall was measured at sites 1, 3, 6, 8, and 9 due to presence of tree species at these sites only. After the end of the month, the deposits were transferred in a washed polyethylene bottle rinsing with deionised water. The solution was stirred and left overnight to obtain the maximum water-soluble ions.

Rainwater samples were collected on an event basis by using a stainless steel funnel of 30-cm diameter fitted on 5-l polyethylene bottles, previously rinsed with deionised water. It has been reported that a stainless steel funnel has no impact on rain

**TABLE 1**
**Characterization of Study Sites**

| Site No. | Sites | Vegetation | Distance from STPP |
|---|---|---|---|
| 1. | Helipad | Plantations of fruit and avenue trees | 2 km N from STPP; 300 m asl |
| 2. | Ashdam | Near Ashdam of STPP; agricultural field; old trees of *Madhuca indica* | 4 km NE from STPP; 225 m asl |
| 3. | Kakari | Near Bina and Kakari open cast coal mines; plantations of avenue trees; agricultural fields | 8 km NE from STPP; 1 km SW from RTPP; 2 km W from coal mines; 225 m asl |
| 4. | Renusagar | Near RTPP residential colony; plantations of avenue trees | 10 km NE from STPP; 2 km SW of ATPP; 220 m asl |
| 5. | Anpara | Near ATPP residential colony; plantations of avenue trees | 14 km NE from STPP; 5 km NE from RTPP; 1 km NE from ATPP; 226 m asl |
| 6. | Karahiya | Agricultural fields; patches of forests | 18 km NE from STPP; 9 km NE from RTPP; 5 km NE from ATPP; 228 m asl |
| 7. | Bairpan | Agricultural fields; dense Sal forests | 23 km NE from STPP; 14 km NE from RTPP; 10 km NE from ATPP; 230 m asl |
| 8. | Kuwari | Forest area | 29 km NE from STPP; 20 km NE from RTPP; 16 km from ATPP; 250 m asl |
| 9. | Control | Forest area | 20 km NW of STPP; 230 m asl |

acidity as rainfall is collected into polyethylene bottles and the funnel only provides a wider surface for collection[30]. The duration of the precipitation sample was 24 h. The contamination of dry deposition cannot be completely ruled out due to long distance of sampling sites. All the rainwater samples were preserved immediately with $CHCl_3$ and were then filtered through Whatman number 41 filter paper and refrigerated at 4°C in the laboratory until completion of the chemical analysis.

### *Chemical Characterization*

All the samples collected as dry and wet depositions were analysed for ionic components such as $NH_4^+$, $NO_3^-$, and $SO_4^{2-}$. $SO_4^{2-}$ content was determined by the turbidimetric method[33]. All the samples, blank and standard, were applied for the same operational conditions such as stirring time, adding $BaCl_2$, stirring speed and measuring turbidity time, etc. $NO_3^-$ content was determined by the phenol disulphamic acid method of Jackson[34], and also by the use of $NO_3^-$ ion electrode with the help of an ion analyzer (Orion EA 940, U.S.). $NH_4^+$ content was determined by the phenate method[35] and also by using an $NH_4^+$ electrode through the ion analyzer (Orion EA 940, U.S.). The pH was measured with the help of a pH electrode standardized with pH 4, 7, and 9.2 reference buffers attached to an ion analyzer (Orion EA 940, U.S.).

### Quality Assurance

Quality control measures were taken to assess contamination and reliability of data. Quality control of the data was performed by comparing the calculated vs. measured conductivity and by cation vs. anion balances of the individual samples. If the differences were less than 10%, the data were treated as reliable. During each ion measurement, blanks were run after five determinations to eliminate the presence of contaminants. The coefficient of variance of replicate analysis was determined for the precision of estimate and variations of less than 10% were found.

## RESULTS

### Air Quality

The concentration of total suspended particulate (TSP) was highest in summer, followed by winter, and were lowest during the rainy season (Table 2). The mean TSP concentration varied from a low of 132 µg m$^{-3}$ at control site 9 to a high of 566 µg m$^{-3}$ at site 3 during summer season. $SO_2$ and $NO_2$ concentrations were higher in winter season followed by summer and lower in rainy season (Table 3). The highest concentration of $SO_2$ was recorded at site 3 near RTPP and the coal mines area. Mean $SO_2$ concentration varied from 34 to 75 µg m$^{-3}$ between sites 1 to 8 and 18 µg m$^{-3}$ at the control site during the winter season. The variations in $NO_2$ concentration did not show a pattern similar to $SO_2$, as maximum $NO_2$ concentration of 50 µg m$^{-3}$ was observed at site 4 near RTPP.

### Variations in Deposition Rate

The deposition rate varied significantly at different sites (Table 4). Dry deposition rate (clearfall and throughfall) was higher during the summer than during the winter season. The dry deposition rate (clearfall) recorded during the summer varied from 0.62 to 2.28 g m$^{-2}$ day$^{-1}$ across the sites. In the summer season, dry deposition (throughfall) at study sites 1, 3, 6, 8, and 9 (control) was 2.29, 3.48, 3.12, 1.51, and 0.82 g m$^{-2}$ day$^{-1}$, respectively.

### Depositional Characteristics

The seasonal average pH value of dry deposition (clearfall) varied from 5.8 to 6.9 during winter and 6.1 to 7.0 during the summer across the sites (Table 5). Throughfall pH varied from 6.3 to 7.1 during the winter and 6.8 to 7.1 in the summer season across the sites. In the rainy season, the mean pH of clearfall wet deposition varied from 6.6 to 7.0 and that of throughfall varied from 6.8 to 7.2 (Table 5).

The seasonal mean concentrations of $NH_4^+$, $NO_3^-$, and $SO_4^{2-}$ ions in dry deposition were highest in summer, followed by winter and then rainy season. The concentrations of all the anions were highest at site 3 and lowest at control site 9. $SO_4^{2-}$ concentration was higher than $NO_3^-$. The mean $NH_4^+$ concentration varied from a low of 0.29 to a high of 1.0 mg l$^{-1}$ in clearfall dry deposition and 0.24 to 0.86 mg l$^{-1}$ in throughfall dry deposition during summer across the sites. The mean $NH_4^+$ concentration in clearfall wet deposition varied from 0.14 to 0.74 mg l$^{-1}$ during the rainy season.

The mean $NO_3^-$ concentration during summer varied from 1.13 to 2.15 mg l$^{-1}$ in clearfall and from 1.26 to 2.56 mg l$^{-1}$ in throughfall dry deposition across the sites. The $NO_3^-$ concentration in wet deposition throughfall was higher than that of clearfall. $SO_4^{2-}$ concentration in the summer season varied from 1.02 to 3.76 mg l$^{-1}$ in clearfall dry deposition and 1.62 to 3.29 mg l$^{-1}$ in throughfall dry deposition. In wet deposition, $SO_4^{2-}$ concentra-

**TABLE 2**
**Seasonal Variations in Mean Concentrations of TSP (µg m$^{-3}$) at Different Sites (Mean values ± 1 SE)**

| Sites | Summer | Rainy | Winter |
|---|---|---|---|
| 1. | 320.33 ± 20 | 184.67 ± 3.5 | 219.00 ± 7.8 |
| 2. | 360.00 ± 8.0 | 200.00 ± 6.5 | 250.00 ± 10 |
| 3. | 566.00 ± 15 | 303.00 ± 10 | 501.70 ± 13 |
| 4. | 397.00 ± 15 | 256.67 ± 8.3 | 334.70 ± 5.6 |
| 5. | 391.00 ± 20 | 242.00 ± 4.3 | 326.30 ± 6.1 |
| 6. | 330.00 ± 5.8 | 222.33 ± 5.0 | 288.00 ± 9.6 |
| 7. | 280.00 ± 8.0 | 200.00 ± 8.0 | 200.00 ± 7.0 |
| 8. | 265.00 ± 17 | 158.33 ± 7.6 | 183.70 ± 4.5 |
| 9. | 132.00 ± 5.3 | 92.33 ± 6.4 | 113.00 ± 3.6 |

**TABLE 3**
**Seasonal Variations in Mean Concentrations of $SO_2$ and $NO_2$ ($\mu g\ m^{-3}$) at Different Sites (Mean values ± 1 SE)**

| | Sulphur Dioxide ($SO_2$) | | | Nitrogen Dioxide ($NO_2$) | | |
|---|---|---|---|---|---|---|
| Sites | Summer | Rainy | Winter | Summer | Rainy | Winter |
| 1. | 37.33 ± 5.04 | 30.00 ± 2.89 | 46.33 ± 3.18 | 30.67 ± 2.96 | 21.33 ± 1.65 | 38.33 ± 3.28 |
| 2. | 42.00 ± 3.20 | 32.80 ± 2.00 | 53.20 ± 6.36 | 27.00 ± 2.89 | 20.00 ± 2.30 | 40.00 ± 2.12 |
| 3. | 61.00 ± 5.86 | 40.00 ± 3.51 | 75.00 ± 2.45 | 38.17 ± 5.31 | 30.00 ± 4.63 | 45.30 ± 3.79 |
| 4. | 47.33 ± 4.98 | 37.50 ± 4.00 | 64.00 ± 4.95 | 45.15 ± 5.00 | 36.00 ± 2.96 | 50.00 ± 4.81 |
| 5. | 42.67 ± 6.36 | 35.33 ± 2.12 | 60.00 ± 6.64 | 32.00 ± 3.46 | 26.00 ± 2.23 | 42.00 ± 1.53 |
| 6. | 50.00 ± 5.00 | 38.67 ± 3.48 | 66.33 ± 5.00 | 30.00 ± 3.21 | 25.67 ± 3.48 | 35.67 ± 2.96 |
| 7. | 33.67 ± 2.33 | 28.00 ± 1.90 | 45.00 ± 4.58 | 28.00 ± 2.30 | 23.00 ± 1.05 | 32.00 ± 1.02 |
| 8. | 30.00 ± 2.00 | 23.33 ± 1.76 | 34.00 ± 2.08 | 25.67 ± 3.28 | 20.67 ± 2.37 | 28.33 ± 1.20 |
| 9. | 16.00 ± 1.15 | 14.64 ± 1.50 | 18.00 ± 1.53 | 14.35 ± 2.33 | 10.00 ± 1.41 | 18.00 ± 1.45 |

**TABLE 4**
**Seasonal Dry Deposition Rates of Clearfall and Throughfall ($g\ m^{-2}\ day^{-1}$) at Different Sites (Mean values ± 1 SE)**

| | Clearfall | | | Throughfall | | |
|---|---|---|---|---|---|---|
| Sites | Summer | Rainy | Winter | Summer | Rainy | Winter |
| 1. | 1.74 ± 0.05 | 0.59 ± 0.04 | 1.08 ± 0.03 | 2.29 ± 0.03 | 1.58 ± 0.03 | 2.13 ± 0.03 |
| 2. | 1.49 ± 0.05 | 0.49 ± 0.02 | 1.22 ± 0.06 | — | — | — |
| 3. | 2.28 ± 0.03 | 1.02 ± 0.05 | 1.92 ± 0.05 | 3.48 ± 0.04 | 1.82 ± 0.02 | 2.73 ± 0.13 |
| 4. | 2.14 ± 0.05 | 0.79 ± 0.02 | 1.73 ± 0.03 | — | — | — |
| 5. | 2.11 ± 0.07 | 0.75 ± 0.04 | 1.58 ± 0.01 | — | — | — |
| 6. | 1.72 ± 0.06 | 0.55 ± 0.02 | 1.46 ± 0.02 | 3.12 ± 0.04 | 1.71 ± 0.02 | 2.38 ± 0.06 |
| 7. | 1.58 ± 0.02 | 0.48 ± 0.02 | 1.35 ± 0.03 | — | — | — |
| 8. | 1.33 ± 0.01 | 0.39 ± 0.04 | 1.06 ± 0.05 | 1.51 ± 0.04 | 1.07 ± 0.04 | 1.21 ± 0.02 |
| 9. | 0.62 ± 0.07 | 0.15 ± 0.02 | 0.44 ± 0.03 | 0.82 ± 0.06 | 0.33 ± 0.01 | 0.68 ± 0.06 |

**TABLE 5**
**Seasonal Variations in pH of Clearfall and Throughfall Depositions at Different Sites (Mean values ± 1 SE)**

| | Clearfall (pH) | | | Throughfall (pH) | | |
|---|---|---|---|---|---|---|
| Sites | Summer | Rainy | Winter | Summer | Rainy | Winter |
| 1. | 6.49 ± 0.07 | 6.64 ± 0.03 | 6.32 ± 0.05 | 7.00 ± 0.10 | 7.07 ± 0.19 | 6.54 ± 0.14 |
| 2. | 6.83 ± 0.03 | 6.66 ± 0.04 | 6.72 ± 0.07 | — | — | — |
| 3. | 6.50 ± 0.03 | 6.56 ± 0.07 | 6.32 ± 0.06 | 6.84 ± 0.19 | 6.81 ± 0.09 | 6.30 ± 0.19 |
| 4. | 6.54 ± 0.01 | 6.69 ± 0.09 | 6.33 ± 0.09 | — | — | — |
| 5. | 6.14 ± 0.06 | 6.69 ± 0.02 | 6.62 ± 0.03 | — | — | — |
| 6. | 6.09 ± 0.04 | 6.74 ± 0.04 | 5.81 ± 0.02 | 6.76 ± 0.06 | 7.22 ± 0.12 | 6.48 ± 0.19 |
| 7. | 6.63 ± 0.07 | 6.75 ± 0.04 | 6.88 ± 0.04 | — | — | — |
| 8. | 6.82 ± 0.04 | 6.91 ± 0.03 | 6.79 ± 0.09 | 7.13 ± 0.05 | 7.10 ± 0.08 | 6.91 ± 0.17 |
| 9. | 7.02 ± 0.02 | 7.04 ± 0.03 | 6.89 ± 0.04 | 7.02 ± 0.02 | 7.06 ± 0.03 | 7.07 ± 0.04 |

**TABLE 6**
**Chemical Constituents of Clearfall and Throughfall Depositions ($mg\ l^{-1}$) at Different Sites (Mean values ± 1 SE)**

| Sites | Chemical Constituents | Summer | Rainy | Winter |
|---|---|---|---|---|
| **Clearfall Depositions** | | | | |
| 1. | $NH_4^+$ | 0.85 ± 0.08 | 0.64 ± 0.15 | 0.69 ± 0.10 |
| | $NO_3^-$ | 1.61 ± 0.12 | 1.33 ± 0.06 | 1.39 ± 0.08 |
| | $SO_4^{2-}$ | 2.77 ± 0.43 | 2.17 ± 0.14 | 2.62 ± 0.08 |
| 2. | $NH_4^+$ | 0.94 ± 0.14 | 0.60 ± 0.02 | 0.81 ± 0.05 |
| | $NO_3^-$ | 1.52 ± 0.11 | 1.44 ± 0.03 | 1.47 ± 0.06 |
| | $SO_4^{2-}$ | 2.81 ± 0.05 | 2.57 ± 0.06 | 2.66 ± 0.15 |
| 3. | $NH_4^+$ | 0.83 ± 0.04 | 0.74 ± 0.07 | 0.78 ± 0.06 |
| | $NO_3^-$ | 2.15 ± 0.23 | 1.77 ± 0.13 | 1.94 ± 0.10 |
| | $SO_4^{2-}$ | 3.76 ± 0.14 | 2.70 ± 0.34 | 3.27 ± 0.16 |
| 4. | $NH_4^+$ | 0.60 ± 0.02 | 0.41 ± 0.01 | 0.50 ± 0.14 |
| | $NO_3^-$ | 1.85 ± 0.01 | 1.65 ± 0.11 | 1.65 ± 0.11 |
| | $SO_4^{2-}$ | 2.37 ± 0.04 | 1.74 ± 0.08 | 2.29 ± 0.07 |
| 5. | $NH_4^+$ | 0.74 ± 0.02 | 0.32 ± 0.06 | 0.63 ± 0.02 |
| | $NO_3^-$ | 1.94 ± 0.02 | 1.68 ± 0.02 | 1.75 ± 0.11 |
| | $SO_4^{2-}$ | 2.87 ± 0.02 | 2.35 ± 0.14 | 2.59 ± 0.06 |
| 6. | $NH_4^+$ | 0.71 ± 0.03 | 0.52 ± 0.06 | 0.76 ± 0.04 |
| | $NO_3^-$ | 2.03 ± 0.33 | 1.82 ± 0.09 | 1.85 ± 0.08 |
| | $SO_4^{2-}$ | 2.94 ± 0.40 | 2.30 ± 0.31 | 2.88 ± 0.08 |
| 7. | $NH_4^+$ | 1.00 ± 0.02 | 0.44 ± 0.05 | 0.99 ± 0.05 |
| | $NO_3^-$ | 1.52 ± 0.09 | 1.22 ± 0.02 | 1.47 ± 0.09 |
| | $SO_4^{2-}$ | 2.35 ± 0.41 | 1.70 ± 0.09 | 1.85 ± 0.06 |
| 8. | $NH_4^+$ | 0.83 ± 0.14 | 0.22 ± 0.03 | 0.63 ± 0.08 |
| | $NO_3^-$ | 1.92 ± 0.06 | 1.68 ± 0.20 | 1.29 ± 0.07 |
| | $SO_4^{2-}$ | 1.78 ± 0.15 | 1.54 ± 0.19 | 1.76 ± 0.17 |
| 9. | $NH_4^+$ | 0.29 ± 0.01 | 0.14 ± 0.02 | 0.13 ± 0.01 |
| | $NO_3^-$ | 1.13 ± 0.06 | 0.81 ± 0.08 | 1.11 ± 0.03 |
| | $SO_4^{2-}$ | 1.02 ± 0.17 | 0.67 ± 0.10 | 0.82 ± 0.10 |
| **Throughfall Depositions** | | | | |
| 1. | $NH_4^+$ | 0.86 ± 0.02 | 0.58 ± 0.03 | 0.71 ± 0.04 |
| | $NO_3^-$ | 1.94 ± 0.01 | 1.37 ± 0.06 | 1.52 ± 0.08 |
| | $SO_4^{2-}$ | 2.62 ± 0.11 | 2.70 ± 0.05 | 2.49 ± 0.03 |
| 3. | $NH_4^+$ | 0.51 ± 0.05 | 0.42 ± 0.01 | 0.37 ± 0.06 |
| | $NO_3^-$ | 2.43 ± 0.03 | 1.86 ± 0.06 | 2.06 ± 0.09 |
| | $SO_4^{2-}$ | 2.93 ± 0.10 | 3.12 ± 0.12 | 2.71 ± 0.03 |
| 6. | $NH_4^+$ | 0.58 ± 0.05 | 0.35 ± 0.02 | 0.89 ± 0.04 |
| | $NO_3^-$ | 2.56 ± 0.09 | 1.83 ± 0.09 | 2.08 ± 0.03 |
| | $SO_4^{2-}$ | 3.29 ± 0.14 | 3.52 ± 0.05 | 3.01 ± 0.08 |
| 8. | $NH_4^+$ | 0.83 ± 0.04 | 0.42 ± 0.03 | 0.86 ± 0.03 |
| | $NO_3^-$ | 1.93 ± 0.03 | 1.43 ± 0.02 | 1.86 ± 0.09 |
| | $SO_4^{2-}$ | 1.95 ± 0.19 | 1.66 ± 0.09 | 1.57 ± 0.04 |
| 9. | $NH_4^+$ | 0.24 ± 0.02 | 0.17 ± 0.01 | 0.22 ± 0.01 |
| | $NO_3^-$ | 1.26 ± 0.09 | 0.70 ± 0.08 | 1.06 ± 0.04 |
| | $SO_4^{2-}$ | 1.62 ± 0.07 | 1.10 ± 0.04 | 1.50 ± 0.05 |

tion varied from 0.64 to 2.78 mg $l^{-1}$ in clearfall and 1.10 to 3.52 mg $l^{-1}$ in throughfall.

## DISCUSSION

The ambient air quality data reveal that pollution load with respect to TSP and dust deposition rate was highest at site 3, which is 8 km NE from STPP and 1 km SW of RTPP. Two open cast coal mines situated at Kakari and Bina are also 2 km W from this site. Among the gases, $SO_2$ also showed the highest concentration at site 3 in all the seasons. $NO_2$, however, showed the highest concentration at site 4, 10 km NE from the STPP in all the seasons. Seasonal changes in the pollution concentration corresponded well with the meteorological conditions. TSP concentration and dust fall rate were higher during summer, whereas $SO_2$ and $NO_2$ concentrations were highest during winter and all of them were lowest during rainy season. Agrawal and Singh[19] and Singh et al.[36] have also reported a similar seasonal pattern of gaseous and particulate pollutants in the area. The mean seasonal TSP concentration during the summer and winter seasons were higher than the acceptable limit set by Central Pollution Control Board (CPCB) India at site 3 only. TSP concentration at site 1 (2 km W,E,N,S of STPP) was lower than at sites 3, 4, 5, and 6. This may be due to the efficient Electro Static Precipitator set at STPP for the reduction of suspended particulates. The highest concentration at site 3 may be ascribed to the fact that site is downwind of STPP as well as in the close proximity of two coal mines. Agrawal and Singh[19] reported TSP concentrations of 795, 401, and 554 µg $m^{-3}$ as compared to the present data of 566, 303, and 501 µg $m^{-3}$ during summer, rainy, and winter seasons, respectively, at site 3. The trend showed that TSP concentration has declined from 1991–1993 to 1999. Similarly $SO_2$ concentration also showed a declining pattern in the area. The highest $SO_2$ concentration recorded during 1992–1993 at site 4 was 233 µg $m^{-3}$ during winter, whereas the present study recorded $SO_2$ concentration to be 75 µg $m^{-3}$. The source of $SO_2$ in the area is mainly the thermal power plants and motor vehicles. $NO_2$ did not show a pattern similar to $SO_2$ during winter as the highest concentration was recorded at site 4, which is very close to RTPP. The higher concentration of $SO_2$ and $NO_2$ during winter may be due to frequent late night and early morning thermal inversion inhibiting vertical mixing and dispersion of pollutants and thus elevating the concentration. The emission rates of $SO_2$ and $NO_2$ were higher also due to higher utilization of coal during winter as compared to summer for same amount of energy. The higher concentration of particulate during summer may also be due to low humidity and higher wind speed. Frequent rainfall during rainy season led to the lowest concentration of all the pollutants in this season.

The dry deposition rate varied significantly during the three seasons both for clearfall and throughfall. Deposition rate was highest during summer and lowest during rainy season. Dust fall rate was also lower during the present study, as compared to that reported by Agrawal and Singh[29] and Singh et al.[36]; the values were 2.28 and 3.06 g $m^{-2}$ $day^{-1}$ during present and earlier recordings, respectively. During the early to midsummer season, the atmospheric conditions remained unstable due to convection and therefore particulate matter from sources and soil origin were lifted up. Because of the dry conditions, there was a higher dry deposition rate. The humid conditions during the end of the summer season tended to maximize deposition rates. The moisturising surface of the canopy and dust favored absorption of gases and particulate. These factors may also account for the greater deposition during the summer season. During rainy season a large flux of moisture is available in the atmosphere, and frequent rainfall led to washout effects on the particulate matter, causing their lower concentration.

The mean monthly pH of the wet deposition indicates that rainfall in the area is not acidic. However, when compared with the control site, the sites closer to the thermal power plants showed a lower range of pH. The variations in pH value clearly show the influence of thermal power plants and open cast coal mines on constituents of rainwater leading to pH changes. The pH of site 3 was lowest. Interestingly, this site showed the highest level of $SO_2$ and SPM throughout the year. The pH of dry deposition was lowest in winter at most of the sites except Bairpan (site 7), when seasonal $SO_2$ and $NO_2$ concentrations were also highest. Khemani et al.[37] reported the lowest pH value at 0.5 km E of STPP and pH increased as distance increased to 1 km. The higher pH of rainwater even at higher emission of acidifying gases $SO_2$ and $NO_2$ may be attributed to the higher alkaline particulate matter, which may have neutralized the rain water acidity. Likens and Bormann[9] have reported neutralizing capacity of fly ash particles released from coal-fired power plants. Subramanian and Sexena[38] have reported the role of particulate matter in maintaining the alkaline rain water pH (7.0 to 8.4) over India including the samples collected near thermal power plants at Delhi. Delhi and Pune, which are urban sites in north and western India, showed pH values of 5.7 and 6.5, respectively, for wet only depositions[39].

Among the sites, $SO_4^{2-}$ concentration was highest at site 3 in all the seasons. Site 3 also had maximum concentration of gaseous $SO_2$. Seasonal variations showed that $SO_4^{2-}$ concentration was highest in the summer season at all sites and lowest during rainy season. At the most polluted site 3, the seasonal variations were not very significant. The sources of $SO_4^{2-}$ particles are from anthropogenic activities in the area. Saxena et al.[40] have reported the highest $SO_4^{2-}$ deposition rate during summer and the lowest during monsoon season. The alkaline nature of the particulate matter would favor the reaction leading to greater adsorption of $SO_2$[41]. Rao et al.[30] have reported $SO_4^{2-}$ dry deposition rate of 5.9 and 5.3 mg $m^{-2}$ $day^{-1}$ in the Pune city from January 1984 to December 1988. The present study clearly shows that even at the control site, dry deposition for $SO_4^{2-}$ was higher than the reported value. Saxena et al.[40], however, reported $SO_4^{2-}$ dry deposition rate (mg $m^{-2}$ $day^{-1}$) from a low of 0.6 during monsoon to 1.8 during summer to a high of 4.6 during winter. This difference in the trend may be ascribed to the difference in the moisture content of the atmosphere at the two places and differences in other meteorological conditions.

$NO_3^-$ deposition was also highest during the summer season as compared to the winter season. The $NO_3^-$ particles formed by the reaction of atmospheric $HNO_3$ with particulates could explain the observed fluxes. Formation of $HNO_3$ is favored due to high day temperature and intense light leading to high concentration of $OH^-$ radicals[42]. Under these conditions the instability of $NH_4^-$ and $NO_3^-$ aerosols may further add to $HNO_3$ concentration[43]. Dry deposited $HNO_3^-$ could be a factor leading to high deposition of $NO_3^-$ flux during summer. Saxena et al.[40] showed a range of $NO_3^-$ dry deposition (mg $m^{-2}$ $day^{-1}$)

from 0.4 in monsoon, 2.6 in summer, and 3.2 in winter in Agra city located at the north central part of India. Rao et al.[30] reported $NO_3^-$ concentration ranging from minimum of 1.13 in monsoon to 1.51 in summer and 1.88 mg $l^{-1}$ in winter at Pune situated in western part of India. $NH_4^+$ concentration was lowest during rainy season and highest in winter season at the most polluted site. $NH_4^+$ dry deposition rates were reported to be 0.07 during summer, 0.02 during monsoon, and 0.11 mg $l^{-1}$ during winter in Pune (1992). The dry deposition fluxes are higher than the wet deposition fluxes.

## CONCLUSION

The present study has pointed out that the wet and dry depositions at various sites are greatly influenced by anthropogenic sources. There was not significant difference in the mean pH of deposition during the dry and wet periods. The low acidic deposition indicates that the anthropogenic sources and natural soil are responsible for emitting particles of alkaline nature, thus neutralizing the acidity. The data indicates that receiving ecosystems are not under threat of acidification under the present scenario, however, strict control of particulate emission from the source may further increase the acidity and atmospheric budget of reactive sulfur and nitrogen oxides in the atmosphere.

## ACKNOWLEDGMENT

The authors thankfully acknowledge the financial assistance of the Ministry of Environment and Forest, Government of India, New Delhi.

| | | |
|---|---|---|
| Received: | October | 24, 2001 |
| Revised: | November | 9, 2001 |
| Accepted: | November | 12, 2001 |
| Published: | December | 19, 2001 |

## BIOSKETCH

**Raj K. Singh** is a Senior Research Fellow at Banaras Hindu University in Varanasi, India. He holds an M.Sc. in Environmental Science and Ecology. His research interests are atmospheric deposition and budgeting of the nutrients and trace elements around industrial sites, air pollution analysis with emphasis on broad-scale patterns in atmospheric wet and dry deposition in tropical deciduous forest region and also characterization and speciation of air pollution chemistry. Dr. Singh is also interested in the role of anthropogenic activities such as industrialization and urbanization on the extent of change in biogeochemical cycles and their effects on soil, plants and microbes.
